# Body Mass Index Status in Italian Children with Celiac Disease at Diagnosis and After 12–18 Months on a Gluten-Free Diet: A Multicenter Retrospective Study

**DOI:** 10.3390/nu18030501

**Published:** 2026-02-02

**Authors:** Alice Monzani, Elena Pozzi, Luisa Abbattista, Marco Crocco, Federica Malerba, Silvia Marcolin, Noemi Paggi, Monica Montuori, Giulia Gagliostro, Claudia Mandato, Angelo Colucci, Fernanda Cristofori, Ruggiero Francavilla, Giovanna Zuin, Sigi Petrela, Francesco Valitutti, Camilla Alberti, Francesco Graziano, Michele Citrano, Simona Spetrino, Elena Lionetti, Andrea Di Siena, Massimo Spina, Chiara Maria Trovato, Barbara Parma, Maurizio Mennini, Naire Sansotta, Enrico Felici, Pier Luigi Calvo, Maria Teresa Illiceto, Chiara Terzi, Federica Ferrari, Licia Pensabene, Lorenza Scotti, Renata Auricchio

**Affiliations:** 1 Division of Pediatrics, Department of Health Sciences, University of Piemonte Orientale, 28100 Novara, Italy; silvia.marcolin@uniupo.it (S.M.); 20016278@studenti.uniupo.it (N.P.); 2 Buzzi Children’s Hospital, 20154 Milan, Italy; elena.pozzi@asst-fbf-sacco.it (E.P.); abbattista.luisa@gmail.com (L.A.); 3 Department of Biomedical and Clinical Sciences, University of Milan, 20157 Milan, Italy; 4 Pediatric Gastroenterology and Endoscopy Unit, Istituto di Ricovero e Cura a Carattere Scientifico Giannina Gaslini, 16147 Genoa, Italy; m.crocco@hotmail.it; 5 Department of Neuroscience, Rehabilitation, Ophthalmology, Genetics, Maternal and Child Health (DINOGMI), University of Genova, 16132 Genoa, Italy; federicamalerba@gaslini.org; 6 Unità Operativa Complessa Pediatria e Neonatologia Imperia, Istituto di Ricovero e Cura a Carattere Scientifico Giannina Gaslini, 18100 Imperia, Italy; 7 Unità Operativa Complessa Gastroenterologia ed Epatologia Pediatrica, Policlinico Universitario Umberto I/Sapienza, 00161 Rome, Italy; m.montuori@policlinicoumberto1.it (M.M.); giulia.gagliostro92@gmail.com (G.G.); 8 Section of Pediatrics, Department of Medicine, Surgery, and Dentistry, “Scuola Medica Salernitana”, University of Salern Baronissi, 84081 Salerno, Italy; cmandato@unisa.it (C.M.); acolucci@unisa.it (A.C.); 9 Interdisciplinary Department of Medicine, Pediatric Section, University of Bari Aldo Moro, 70126 Bari, Italy; fernandacristofori@gmail.com (F.C.); rfrancavilla@gmail.com (R.F.); 10 Pediatric Department, Fondazione Istituto di Ricovero e Cura a Carattere Scientifico San Gerardo dei Tintori, 20900 Monza, Italy; giovanna.zuin@irccs-sangerardo.it (G.Z.); s.petrela@campus.unimib.it (S.P.); 11 Pediatrics Section, Department of Medicine and Surgery, University of Perugia, 06129 Perugia, Italy; francesco.valitutti@unipg.it (F.V.); camilla.alberti@hotmail.it (C.A.); 12 Pediatric Unit, Villa Sofia—Cervello Hospital, 90146 Palermo, Italy; fra.graziano23@gmail.com (F.G.); citranomichele@gmail.com (M.C.); 13 Department of Translational Medical Sciences, University of Naples Federico II, 80131 Naples, Italyr.auricchio@unina.it (R.A.); 14 Department of Specialized Clinical Sciences and Odontostomatology, Polytechnic University of Marche, 60126 Ancona, Italy; m.e.lionetti@staff.univpm.it; 15 Division of Pediatrics, Department of Medicine, Santa Maria della Misericordia University Hospital, 33100 Udine, Italy; disienaandrea@gmail.com; 16 Dipartimento di Medicina Clinica e Sperimentale, University of Catania, 95123 Catania, Italy; massim.spina@tiscali.it; 17 Unità Operativa Semplice Riabilitazione Nutrizionale, Unità Operativa Complessa Gastroenterologia e Nutrizione, Ospedale Pediatrico Bambino Gesù, Istituto di Ricovero e Cura a Carattere Scientifico, 00165 Rome, Italy; chiaramariatrovato@gmail.com; 18 Pediatric Department, “Mariani” Center for Fragile Child, Azienda Socio-Sanitaria Territoriale Lariana, Sant’Anna Hospital, 22042 Como, Italy; barbaraparma79@hotmail.com; 19 Dipartimento di Neuroscienze, Salute Mentale e Organi di Senso (NESMOS), U.O.C. Pediatria, Azienda Ospedaliera Universitaria Sant’Andrea, Sapienza Università di Roma, 00189 Rome, Italy; maurizio.mennini@gmail.com; 20 Paediatric Hepatology Gastroenterology and Transplantation, Papa Giovanni XXIII Hospital, 24127 Bergamo, Italy; nsansotta@asst-pg23.it; 21 Pediatric and Pediatric Emergency Unit, Children Hospital, Azienda Ospedaliero-Universitaria SS. Antonio e Biagio e C. Arrigo, 15121 Alessandria, Italy; enrico.felici@ospedale.al.it; 22 Pediatric Gastroenterology Unit, Department of Pediatrics, Azienda Ospedaliero Universitaria Città della Salute e della Scienza di Torino, Ospedale Infantile Regina Margherita, 10126 Turin, Italy; pierluigi.calvo@unito.it; 23 Pediatric Gastroenterology and Digestive Endoscopic Unit, Department of Pediatrics, “Santo Spirito” Hospital of Pescara, 65124 Pescara, Italy; mtilli600@yahoo.it; 24 Pediatric Unit, Bolognini Hospital, Seriate, 24068 Bergamo, Italy; chiara.terzi@asst-bergamoest.it; 25 Pediatric Unit, Sant’Eugenio Hospital, 00144 Rome, Italy; federicaferrari.1985@gmail.com; 26 Pediatric Unit, Department of Medical and Surgical Sciences, University Magna Graecia of Catanzaro, 88100 Catanzaro, Italy; pensabene@unicz.it; 27 Department of Translational Medicine, University of Piemonte Orientale, 28100 Novara, Italy; lorenza.scotti@uniupo.it

**Keywords:** pediatric, children, celiac disease, gluten-free diet, body mass index, underweight, overweight, obesity

## Abstract

**Background/Objectives**: The distribution of body mass index (BMI) categories at celiac disease (CD) diagnosis in children is changing, and the impact of a gluten-free diet (GFD) on BMI status remains incompletely understood. We aimed to evaluate the distribution of BMI categories at CD diagnosis and their changes after 12–18 months on a GFD in Italian children. **Methods**: Children and adolescents aged 0–18 years who received a new diagnosis of CD at 23 Pediatric Gastroenterology referral centers in Italy were retrospectively enrolled. We analyzed their BMI status at diagnosis, classifying them as underweight, normal weight, overweight, or obese. BMI changes were assessed after 12–18 months on a GFD. **Results**: Among the 4967 children (mean age 7.1 ± 4.1 years, M:F = 1827:3140), 4.4% were underweight, 77.5% normal weight, 12.7% overweight, and 5.4% obese at diagnosis. Overweight/obese children were more likely to have a family history of CD, associated conditions, and an asymptomatic presentation. After 12–18 months of GFD, 55.7% of underweight children achieved normal weight, and 23% of overweight/obese reverted to normal weight. Conversely, 10.9% of normal-weight children and 3.2% of underweight children became overweight/obese. **Conclusions**: At diagnosis, most children were normal weight, but 18.1% presented with overweight/obesity. After 12–18 months on a GFD, BMI normalized in over half of underweight but in fewer than one-quarter of overweight/obese subjects.

## 1. Introduction

Historically, celiac disease (CD) in children has been associated with malabsorption-related phenotypes and underweight, with overweight and obesity considered rare presentations [1]. However, over the past two decades, there has been a substantial shift in the clinical presentation of CD, with increasing numbers of children presenting with normal weight or even overweight/obesity at diagnosis, often with atypical or asymptomatic forms. As early as 2014, a large cohort study in Swedish children concluded that, although children with CD generally have lower body mass index (BMI) compared to their non-CD peers at a population level, growth parameters alone are unreliable for individual diagnosis [2].

Over the years, the prevalence of CD children presenting with overweight/obesity has increased worldwide. A recent meta-analysis by Barone et al. [3] reported a pooled prevalence of 14% (95%CI, 11–17%) for overweight and 6% (95%CI, 4–8%) for obesity in pediatric patients. Country-specific data also show considerable variability: 19% and 16% in two North American cohorts [4,5], 13.9% in Israel [6], 13.2% in Italy [7], and 9.1% in India [8].

The impact of a strict, lifelong gluten-free diet (GFD)—the cornerstone of CD management—on BMI has been investigated in pediatric populations, with conflicting results. GFD induces mucosal healing and nutritional repletion, which may promote weight gain, a desired outcome in underweight patients. Conversely, excessive weight gain could increase the risk of overweight and obesity [3]. In addition, inflammation—both as a hallmark of obesity and as a consequence of gluten exposure in untreated CD—may contribute to these outcomes [9]. Findings from previous studies are inconsistent: while some report BMI normalization in underweight children after GFD initiation, others describe a general tendency toward weight gain. Overall, BMI tends to increase after diagnosis, though this does not necessarily translate into an increased risk of overweight or obesity [3].

Given these considerations, understanding the trajectory of BMI changes in children with CD and identifying clinical characteristics associated with different BMI categories at diagnosis is increasingly important. Such knowledge can guide personalized follow-up strategies and nutritional counseling after diagnosis.

A country-specific analysis is particularly relevant in Italy, where dietary habits, cultural approaches to food, and the long-standing availability and reimbursement of gluten-free products may uniquely influence nutritional behaviors and BMI trajectories in children with celiac disease, and should therefore be taken into account when interpreting findings from other populations.

This study aimed to assess the distribution of BMI categories at diagnosis in a cohort of Italian children with CD, compare clinical characteristics between underweight/normal weight and overweight/obese subjects, and evaluate changes in BMI status after 12–18 months on a GFD.

## 2. Materials and Methods

### 2.1. Study Design and Sampling

This was a national multicenter retrospective study with both cross-sectional and longitudinal components, including children and adolescents aged 0–18 years who received a new diagnosis of CD between 1 March 2013, and 1 March 2023, at 23 pediatric gastroenterology referral centers in Italy.

Subjects were eligible for enrollment if anthropometric data (weight and height) at diagnosis were available and if they had a confirmed diagnosis of CD according to current ESPGHAN guidelines [10,11] during the 10-year study period. Patients were followed from the date of diagnosis up to 12–18 months on a GFD.

The study was conducted in accordance with local Ethics Committee regulations, the Declaration of Helsinki, and Good Clinical Practice guidelines. Written informed consent was obtained from all parents and, when appropriate, from the patients themselves. The study protocol was approved by the local Ethics Committees (CE015/2024).

### 2.2. Definition of BMI Categories

First, BMI was calculated as body weight divided by height squared (kg/m^2^). BMI z-scores were calculated with the LMS method [12], which takes into account skewness for the deviation from a normal distribution using a Box–Cox transformation (L), median (M), and coefficient of variation (S), considering the gender- and age-specific reference values derived from the international (International Obesity Task Force; IOTF) body mass index (BMI) cut-offs [13]. Study subjects were categorized into: underweight (UW, BMI < 3rd percentile), normal weight (NW, BMI 3rd–75th percentile), overweight (OW, BMI 75th–95th percentile), and obesity (OB, BMI > 95th percentile). BMI categories were assessed at diagnosis and after 12–18 months on a GFD.

### 2.3. Assessment of Factors Related to BMI Categories

At diagnosis, demographic, anthropometric, and clinical characteristics were collected, as well as modality of CD diagnosis and serological data. Specifically, the following information were retrieved: sex, age at diagnosis, measured height and weight, the main presenting symptom (recurrent abdominal pain, diarrhea, constipation, failure to thrive, anemia, fatigue, headache, dermatitis, other symptoms or no symptoms referred); the presence of one or more first-degree relative with CD; the concomitant diagnosis of other CD-related diseases (e.g., autoimmune thyroiditis, type 1 diabetes), CD diagnosis with or without biopsy and maximum anti-tissue transglutaminase IgA antibodies (anti-tTG) titer, expressed as number of times the upper limit of normal (× ULN) for the reference laboratory.

In analyzing the main presenting symptoms, failure to thrive was considered separately from other symptoms as it was expected to be differently distributed according to different BMI classes.

If available, height and weight after 12–18 months on a GFD were also collected.

### 2.4. Statistical Analysis

Descriptive statistics were used to summarize the demographic, anthropometric and clinical characteristics collected on the study sample. Categorical variables were reported as absolute frequencies and percentages while numerical variables as mean and standard deviation (SD) or median and interquartile range (IQR) if not distributed as a normal random variable according to the Shapiro–Wilk test and the analysis of QQ plot. The descriptive statistics were calculated on the overall sample and stratified for BMI classes (UW, NW, OW, OB). Chi square, analysis of variance (ANOVA) and Kruskall–Wallis tests were used to assess the relationship between the patients’ characteristics and BMI classes. Missing data were handled using a complete-case analysis approach, since these data were missing completely at random, as unrelated to any patient characteristics. This approach was considered appropriate given the large sample size and the low rate of missingness. All patient characteristics considered in the study were included in the multivariable models. Moreover, univariable and multivariable nominal logistic regression models were applied to estimate the prevalence odds ratios (pORs) and adjusted prevalence odd ratios (apORs) and the corresponding 95% confidence interval (95%CI) for the association between the patients’ characteristics and BMI classes. Multinomial logistic regression was chosen over ordinal logistic regression since the aim was to allow for separate estimation of the association between covariates and each weight category, using NW as the reference group. Finally, McNemar’s test was used to assess the presence of a change in the BMI categories from diagnosis to 12–18 months on a GFD. For all statistical tests, the type I error was set at 0.05. The analyses were performed using SAS v9.4 [SAS Institute, Cary, NC, USA].

## 3. Results

**Figure 1:**
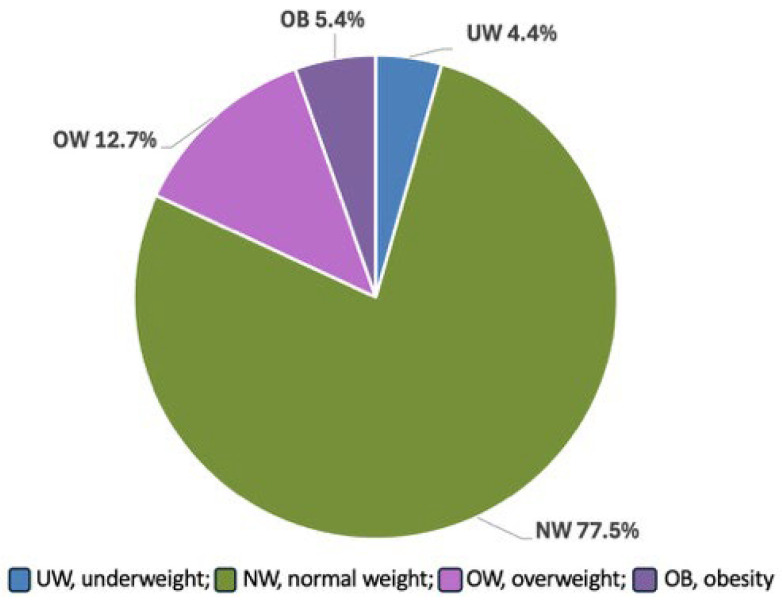
BMI class distribution in Italian children at CD diagnosis.

Overall, 4967 Italian children were included in the study. Figure 1 shows the distribution of BMI classes at diagnosis. A total of 219 children were UW (4.4%), 3847 (77.5%) were NW, 633 (12.7%) OW, and 268 (5.4%) OB, respectively.

Table 1 presents the descriptive statistics for the cohort overall and stratified by BMI category.

At diagnosis, 3791 children (76.3%) reported at least one symptom. OB patients were older than UW and NW ones (*p* < 0.0001 and *p* = 0.025, respectively) and OW children were older than NW peers (*p* < 0.0001). Family history of CD (*p* = 0.001), CD-related diseases (*p* < 0.0001), biopsy-sparing diagnoses (*p* = 0.001) and asymptomatic disease (*p* < 0.0001) were associated with BMI status. Specifically, the prevalence of family history for CD (33.8% OW/OB vs. 29.4% NW and 24.8% UW), CD-related diseases (14.8% vs. 8.9% and 8.2%), and asymptomatic disease (32.1% vs. 22% and 18.3%) were higher in children with an increased BMI status while the prevalence of biopsy-sparing diagnoses (52.2% vs. 58.7% and 61.6%) was lower compared to their UW and NW peers. Median anti-tTG (expressed as × ULN) were lower in OW and OB subjects compared to UW and NW ones.

**Figure 2:**
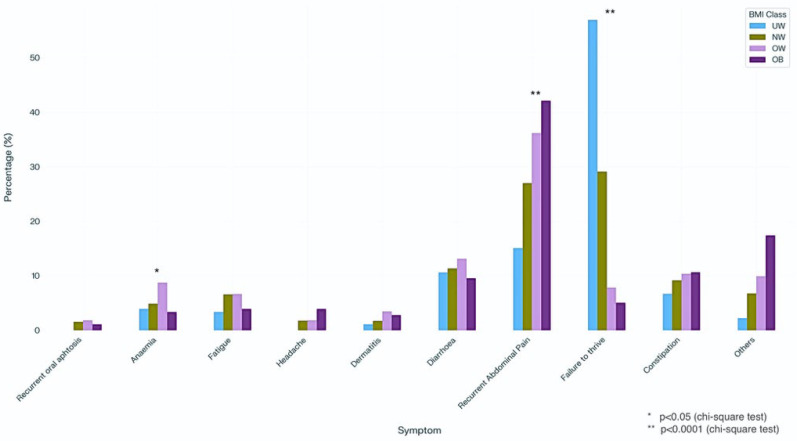
Frequency distribution of the main presenting symptoms at CD diagnosis.

Figure 2 shows the frequency distribution of the main presenting symptoms.

Among the UW children, failure to thrive was the most common symptom, followed by recurrent abdominal pain and diarrhea. In NW children, failure to thrive—although they were of normal weight—was reported in nearly one-third of cases, followed by recurrent abdominal pain and diarrhea/constipation. In OW children, recurrent abdominal pain was the main presenting symptom, followed by diarrhea and constipation, while symptomatic OB children reported abdominal pain predominantly. Interestingly, failure to thrive was reported in 7.8% of OW and 5.1% of OB children.

Table 2 reports the results of the univariable and multivariable multinomial logistic regression models.

The results of the multivariable model show that sex, diagnostic approach, and geographic location remained significantly associated only with OB (Sex—apOR 2.03; 95%CI 1.55–2.66; diagnosis by duodenal biopsies vs. biopsy-sparing—apOR 1.37; 95%CI 1.03–1.82; Central vs. Northern Italy—apOR 1.92; 95%CI 1.35–2.74). The associations of age, CD-related diseases, and failure to thrive with BMI categories remained consistent, although the apORs were slightly lower than in the univariate model.

Follow-up data after 12–18 months (median 13 months, IQR: 12–16 months) of GFD were available for 3679 subjects. Among them 2972 (80.78%) did not change their BMI status, 235 (6.39%) decreased their BMI, and 472 (12.82%) increased it over this short time period (*p* < 0.0001) (Table 3).

**Figure 3:**
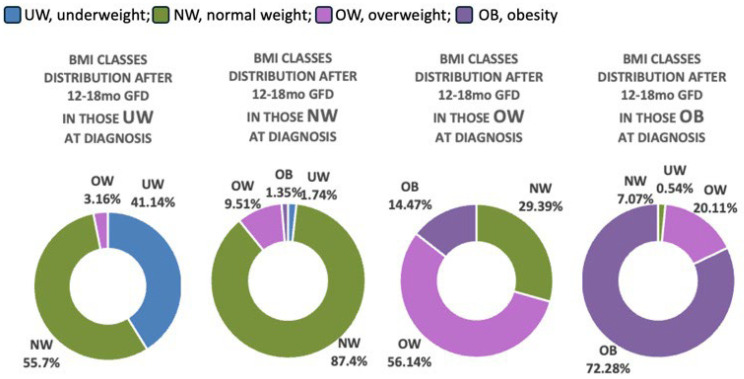
Frequency distribution of BMI classes after 12–18 of GDF stratified according to the classes of BMI at baseline.

Changes in BMI class distribution according to BMI status at diagnosis are shown in Figure 3.

## 4. Discussion

The primary aim of this multicenter study was to describe the distribution of BMI categories at the time of CD diagnosis in an Italian pediatric population. In line with recent international reports [3], most patients were classified as normal weight (NW). Nevertheless, 4.4% of children were underweight (UW), while more than 18% presented with overweight (OW) or obesity (OB), a pattern that contrasts with the historically malnourished phenotype of CD.

Several hypotheses have been proposed to explain the coexistence of CD with overweight/obesity. The most widely accepted is the compensatory theory, which suggests that unaffected intestinal segments adapt to mucosal damage by increasing absorptive capacity [14]. Structural adaptations such as villous elongation, crypt hypertrophy, and increased enterocyte numbers have been described and could theoretically contribute to enhanced nutrient absorption. However, given the retrospective and observational nature of the available data, a causal relationship between these adaptive mechanisms and excess weight could not be established. In our cohort, children with overweight or obesity were older than their normal-weight peers. One possible explanation is that adaptive intestinal changes may become more pronounced with age; however, this interpretation remains speculative. Alternatively, older age at diagnosis may reflect a delayed recognition of celiac disease in children with OW/OB, who are more often oligo- or asymptomatic and therefore more likely to be diagnosed through active case finding rather than symptom-driven evaluation. As the duration of symptoms before diagnosis was not assessed in this study, this hypothesis should be interpreted with caution and warrants further investigation in prospective studies.

Family history of CD, presence of CD-related comorbidities, and asymptomatic presentation were all more frequent among OW/OB children, while failure to thrive remained strongly associated with UW status and, surprisingly, was also a common complaint among NW children. The higher prevalence of asymptomatic disease in OW/OB children raises concerns that CD may be underrecognized or diagnosed late in this group, as clinicians may not initially suspect CD in the absence of classical symptoms or poor growth. When symptomatic, abdominal pain was the leading complaint in children with excess BMI, consistent with previous studies [4,15]. The higher prevalence of autoimmune comorbidities among OW/OB patients is in line with the known link between excess weight and autoimmunity, likely mediated by adipokines [16].

In our series, OW and OB subjects were more likely to be diagnosed via duodenal biopsy rather than biopsy-sparing criteria. This may partly reflect the study’s time frame, which included years before the widespread adoption of the 2020 ESPGHAN guidelines [11], when biopsy-sparing diagnosis was restricted to symptomatic patients. Lower median anti-tTG titers in OW/OB children may also have reduced the likelihood of meeting non-biopsy diagnostic criteria, necessitating histological confirmation.

An unexpected finding in our study was the male predominance in the OW/OB group. Although this contrasts with the well-known female predominance in CD overall, it aligns with national epidemiological data on pediatric obesity in Italy (https://www.epicentro.iss.it/okkioallasalute/, accessed on 4 January 2026), where boys are more frequently affected.

Multinomial logistic regression confirmed that male sex, older age, presence of CD-related comorbidities, and diagnosis by duodenal biopsy were associated with increased odds of OW/OB status compared with NW. Regional differences also emerged, with children from Central Italy more likely to be obese than those from Northern regions. Potential regional differences observed in our cohort may reflect variations in lifestyle and dietary patterns across Italy. However, as socioeconomic variables were not directly collected, these hypotheses could not be formally tested within the present study. Differences in access to healthcare services are unlikely, given the universal coverage of the Italian National Health System, but this aspect was not specifically evaluated and warrants further investigation.

Failure to thrive was a strong predictor of being underweight and inversely associated with OW/OB, reinforcing its diagnostic value for identifying malnourished children with CD. Conversely, asymptomatic children were more likely to be overweight/obese, which underscores the importance of proactive case-finding strategies in at-risk populations, especially among those with a family history of CD or related comorbidities [4,17]. An apparently paradoxical finding of our study was that a proportion of patients classified as overweight/obese were nonetheless referred with a complaint of “failure to thrive”. This reflects the way data were collected, as only the main reason for referral was recorded, which may capture parental perception more than objective anthropometric evaluation. In some cases, “failure to thrive” may have been used to indicate impaired statural growth or a period of weight stabilization, rather than underweight. This highlights the potential discrepancy between subjective concerns reported at referral and the actual growth status documented at diagnosis.

When examining BMI trajectories after 12–18 months on a GFD, more than half of UW patients normalized their BMI, whereas only 29% of OW and 7% of OB children reverted to normal weight. These findings indicate that catch-up growth in underweight individuals may occur earlier than BMI reduction in children with excess weight. In the latter group, weight normalization appears to be slower and may reflect a more complex and multifactorial process, potentially influenced by age and other unmeasured factors. Longer follow-up and prospective studies are needed to better clarify these trajectories and their underlying mechanisms.

Notably, nearly 2% of children that were NW at diagnosis progressed to UW. This tendency, already described in a smaller Italian cohort [18], may suggest that gluten avoidance can lead to excessive food selectivity, resulting in inadequate intake and subsequent weight loss or insufficient weight gain. Similarly, a high rate (up to 40%) of children with food allergies was reported to have feeding difficulties, described as suboptimal intake of food and/or lack of age-appropriate eating habits [19]. This possibility should be kept in mind, and if growing trajectories tend to decline in NW subjects after the diagnosis, appropriate and timely dietary—and in some cases, psychological—support should be provided. Conversely, 11% of NW and about 3% of UW children transitioned into OW/OB categories after starting the GFD, consistent with previous studies [4,5]. This aligns with a recent systematic review and meta-analysis [3], which confirmed a general tendency toward weight gain after CD diagnosis. However, this should not be interpreted as an inherent risk of the GFD itself, but rather because of unbalanced dietary choices, particularly the overuse of highly processed gluten-free products with high glycemic and lipid indices [9,20,21]. Nutritional counseling should therefore not only emphasize strict gluten exclusion but also promote balanced, age-appropriate diets [22]. Indeed, Reilly et al. [4] reported that two-thirds of obese children reduced their BMI z-scores after GFD, while all non-compliant OW patients increased their BMI at follow-up, suggesting that adherence to GFD often coincides with overall healthier dietary habits.

Despite its strengths, this retrospective observational study has some limitations. First, compliance with the GFD was not systematically assessed, which restricts our ability to attribute BMI status changes solely to dietary factors. It is plausible that differences in adherence to the diet among children belonging to different initial BMI classes may have contributed to the observed trajectories. Moreover, even if one assumes an equal distribution of GFD compliance across groups, we did not evaluate dietary habits or diet composition, which could have helped to clarify whether children who developed overweight/obesity were more likely to adopt an obesogenic dietary pattern, independent of gluten exclusion. The lack of these data reflects the retrospective nature of our study and the heterogeneity of clinical practice among participating centers. Future prospective studies should incorporate the standardized assessment of both GFD adherence and dietary quality to better disentangle their role in shaping growth and nutritional outcomes in children with CD.

Second, information on associated autoimmune disorders (such as type 1 diabetes or autoimmune thyroiditis) was available only at diagnosis. Although these conditions may influence growth and nutritional status, we could not evaluate their impact during follow-up due to the lack of longitudinal data about their possible onset after CD diagnosis. Moreover, we were not able to assess whether BMI changes were associated with antibody persistence or negativization during follow-up, as serological data were not consistently available across participating centers. Future studies including both anthropometric and serological follow-up parameters could clarify this potential relationship.

Third, physical activity was not evaluated. Although it is unlikely that the children changed their activity levels substantially before and after diagnosis, differences in activity could have influenced the BMI overall. Future studies should incorporate objective measures such as accelerometry, as self-reported questionnaires are often unreliable.

Finally, inflammatory markers and appetite-regulating hormones (e.g., leptin, ghrelin), which might play a role in BMI trajectories, were not measured.

Further longitudinal studies incorporating these parameters are necessary to better understand the multifactorial drivers of BMI status evolution in pediatric CD and to optimize dietary management accordingly.

## 5. Conclusions

In conclusion, this large multicenter study shows that in contrast to the historically malnourished phenotype, a substantial proportion of Italian children with CD present with overweight/obesity at diagnosis. BMI status was associated with demographic, clinical, and regional factors, highlighting the complexity of nutritional outcomes in CD. After 12–18 months of GFD, more than half of the UW children normalized their BMI, while fewer OW/OB children reverted to normal weight, indicating that early catch-up growth occurs more readily than weight reduction.

These findings underscore the importance of considering CD in the differential diagnosis, even in children with overweight/obesity and highlight the need for personalized follow-up strategies. Nutritional counseling should go beyond gluten exclusion to ensure balanced diets, prevent both undernutrition and overweight/obesity, and support optimal growth trajectories. Future prospective, harmonized multicenter studies are needed to clarify long-term patterns and mechanisms underlying BMI evolution in pediatric CD.

## Figures and Tables

**Table 1:** Demographic and clinical characteristics of the enrolled subjects overall and by BMI classes, at diagnosis.

	UW N = 219	NW N = 3847	OW N = 633	OB N = 268	Total N = 4967	Chi Square Test
	N (%)	N (%)	N (%)	N (%)	N (%)	*p*-Value
**Sex**						
Females	130 (59.36)	2500 (64.99)	385 (60.82)	125 (46.64)	3140 (63.22)	<0.0001
Males	89 (40.64)	1347 (35.01)	248 (39.18)	143 (53.36)	1827 (36.78)	
**Age, mean (SD)**	7.35 (4.53)	6.91 (4.05)	7.68 (3.96)	8.38 (3.68)	7.10 (4.06)	<0.0001 *
**Ethnic origin**						
Non-Italian	15 (7.08)	159 (4.34)	17 (2.83)	10 (3.94)	201 (4.25)	0.0635
Italian	197 (92.92)	3502 (95.66)	584 (97.17)	244 (96.06)	4527 (95.75)	
Missing	7	186	32	14	239	
**Geographic location**						
Northern Italy (10 centers)	106 (48.40)	1829 (47.54)	283 (44.71)	110 (41.04)	2328 (46.87)	0.2372
Central Italy (7 centers)	45 (20.55)	881 (22.90)	158 (24.96)	76 (28.36)	1160 (23.35)	
Southern Italy (6 centers)	68 (31.05)	1137 (29.56)	192 (30.33)	82 (30.60)	1479 (29.78)	
**Diagnosis modality**						
Biopsy-sparing	135 (61.64)	2259 (58.82)	338 (53.40)	132 (49.25)	2864 (57.66)	0.0013
By duodenal biopsies	84 (38.36)	1588 (41.28)	295 (46.60)	136 (50.75)	2103 (42.34)	
**anti-tTG** × **ULN,** **median (Q1–Q3)**	18.29 (6.08–55.49)	15.89 (9.86–37.17)	12.90 (7.90–25.56)	10.40 (5.70–26.50)	15.00 (9.00–35.25)	<0.0001 ^
Missing	3	99	18	13	133	
**Family history for CD**						
No	158 (75.24)	2565 (70.56)	392 (66.55)	165 (65.22)	3280 (69.98)	0.0247
Yes	52 (24.76)	1070 (29.44)	197 (33.45)	88 (34.78)	1407 (30.02)	
Missing	9	212	44	15	280	
**CD-related diseases**						
No	191 (91.83)	3311 (91.11)	510 (86.00)	213 (83.20)	4225 (90.07)	<0.0001
Yes	17 (8.17)	323 (8.89)	83 (14.00)	43 (16.80)	466 (9.93)	
Type I diabetes °	2 (11.76)	62 (19.20)	18 (21.69)	8 (18.60)	90 (19.31)	0.8161
Thyroid disease °	2 (11.76)	68 (21.05)	19 (22.89)	10 (23.26)	99 (21.24)	0.7629
Others °	13 (76.47)	193 (59.75)	46 (55.42)	25 (58.14)	277 (59.44)	0.4498
Missing	11	213	40	12	276	
**Clinical presentation**						
Without symptoms	40 (18.26)	847 (22.02)	199 (31.44)	90 (33.58)	1176 (23.68)	<0.0001
Failure to thrive	102 (46.58)	874 (22.72)	34 (5.37)	9 (3.36)	1019 (20.52)	
Other symptoms	77 (35.16)	2126 (55.26)	400 (63.19)	169 (63.06)	2772 (55.81)	

UW, underweight; NW, normal weight; OE, overweight; OB, obesity; anti-tTG, IgA antibodies anti-tissue transglutaminase; × ULN, number of times the upper limit of normal; CD, celiac disease, * ANOVA test, ^ Kruskall–Wallis test, ° percentages calculated among subjects with CD-related diseases.

**Table 2:** Prevalence odds ratios (pORs) and corresponding 95% confidence intervals for the association between subjects’ demographic and clinical characteristics and BMI classes derived from univariable and multivariable nominal logistic regression.

	Univariable Model				Multivariable Model			
	UW	NW	OW	OB	UW	NW	OW	OB
	pOR (95%CI)	pOR (95%CI)	pOR (95%CI)	pOR (95%CI)	apOR (95%CI)	apOR (95%CI)	apOR (95%CI)	apOR (95%CI)
**Sex** M vs. F	1.27 (0.96–1.68)	1 (ref)	**1.20** **(1.01–1.42)**	**2.12** **(1.66–2.72)**	1.14 (0.85–1.53)	1 (ref)	1.18 (0.97–1.42)	**2.03** **(1.55–2.66)**
**Age**	1.03 (0.99–1.06)	1 (ref)	**1.05** **(1.03–1.07)**	**1.09** **(1.06–1.12)**	**1.06** **(1.02–1.10)**	1 (ref)	**1.03** **(1.00–1.05)**	**1.07** **(1.04–1.11)**
**Italian origin** No vs. yes	1.68 (0.97–2.90)	1 (ref)	0.64 (0.39–1.07)	0.90 (0.47–1.73)	1.61 (0.89–2.91)	1 (ref)	0.61 (0.36–1.04)	0.94 (0.48–1.85)
**Diagnosis** by duodenal biopsies vs. Biopsy-sparing	0.89 (0.67–1.17)	1 (ref)	**1.24** **(1.05–1.47)**	**1.47** **(1.14–1.88)**	0.75 (0.55–1.02)	1 (ref)	1.11 (0.92–1.35)	**1.37** **(1.03–1.82)**
**Family history for CD** Yes vs. No	0.79 (0.57–1.09)	1 (ref)	1.21 (1.00–1.45)	1.28 (0.98–1.67)	0.84 (0.59–1.18)	1 (ref)	1.10 (0.90–1.35)	1.15 (0.86–1.55)
**CD-related diseases** Yes vs. No	0.91 (0.55–1.52)	1 (ref)	**1.67** **(1.29–2.16)**	**2.07** **(1.46–2.93)**	0.88 (0.52–1.51)	1 (ref)	**1.53** **(1.16–2.03)**	**1.74** **(1.20–2.54)**
**Geographic location**								
Central vs. Northern Italy	0.88 (0.62–1.26)	1 (ref)	1.16 (0.94–1.43)	**1.44** **(1.06–1.94)**	0.8 (0.53–1.21)	1 (ref)	1.25 (0.97–1.63)	**1.92** **(1.35–2.74)**
Southern vs. Northern Italy	1.03 (0.75–1.41)	1 (ref)	1.09 (0.9–1.33)	1.20 (0.89–1.61)	1.03 (0.73–1.46)	1 (ref)	1.10 (0.88–1.39)	1.33 (0.95–1.87)
**Symptoms**								
Failure to thrive vs. No	**2.47** **(1.69–3.61)**	1 (ref)	**0.17** **(0.11–0.24)**	**0.10** **(0.05–0.19)**	**2.84** **(1.87–4.31)**	1 (ref)	**0.17** **(0.11–0.26)**	**0.12** **(0.06–0.25)**
Other symptoms vs. No	0.77 (0.52–1.13)	1 (ref)	**0.80** **(0.66–0.97)**	**0.75** **(0.57–0.98)**	0.78 (0.51–1.20)	1 (ref)	0.88 (0.70–1.09)	0.83 (0.61–1.14)

UW, underweight; NW, normal weight; OE, overweight; OB, obesity; apOR: adjusted prevalence odds ratio. Values in bold are the statistically significant ones.

**Table 3:** Distribution and transitions of BMI categories from the time of celiac disease diagnosis to 12–18 months after starting a gluten-free diet (GFD).

		BMI Status at 12–18 Months on a GFD				
		UW	NW	OW	OB	Total
**BMI status at diagnosis**	**UW**	65 (41.14)	88 (55.70)	5 (3.16)	0 (0.00)	158
	**NW**	50 (1.74)	2518 (87.40)	274 (9.51)	39 (1.35)	2881
	**OW**	0 (0.00)	134 (29.39)	256 (56.14)	66 (14.47)	456
	**OB**	1 (0.54)	13 (7.07)	37 (20.11)	133 (72.28)	184
	**Total**	116	2753	572	238	3679

UW, underweight; NW, normal weight; OE, overweight; OB, obesity.

## Data Availability

The raw data supporting the conclusions of this article will be made available by the authors on request.

## Abbreviations

The following abbreviations are used in this manuscript:

BMI	Body mass index
CD	Celiac disease
GFD	Gluten-free diet
UW	Underweight
NW	Normal weight
OW	Overweight
OB	Obese
TG	Transglutaminase
ULN	Upper limit of normal
